# Interventional Radiology for High-Flow Aneurysm of the Pancreaticoduodenal Arcades with Median Arcuate Ligament Syndrome: Review of 14 Patients

**DOI:** 10.3390/jcm12144692

**Published:** 2023-07-14

**Authors:** Valentin Siauve, Olivier Chevallier, Amin Mazit, Nicolas Falvo, Pierre-Olivier Comby, Romaric Loffroy

**Affiliations:** 1 Department of Vascular and Interventional Radiology, Image-Guided Therapy Center, François-Mitterrand University Hospital, 14 Rue Paul Gaffarel, BP 77908, 21079 Dijon, France; valentin.siauve@gmail.com (V.S.); olivier.chevallier@chu-dijon.fr (O.C.); amin.mazit@chu-dijon.fr (A.M.); nicolas.falvo@chu-dijon.fr (N.F.); 2 ICMUB Labboratory, UMR CNRS 6302, University of Burgundy, 9 Avenue Alain Savary, 21000 Dijon, France; pierre-olivier.comby@chu-dijon.fr; 3 Department of Neuroradiology and Emergency Radiology, François-Mitterrand University Hospital, 14 Rue Paul Gaffarel, BP 77908, 21079 Dijon, France

**Keywords:** celiac trunk, high-flow aneurysm, interventional radiology, median arcuate ligament, pancreaticoduodenal artery, transcatheter arterial embolization

## Abstract

Transarterial embolization (TAE) for high-flow pancreaticoduodenal artery (PDA) aneurysms in patients with celiac-trunk stenosis by the median arcuate ligament (MAL) has been found effective both after rupturing and to prevent rupture. The objective was to describe the TAE techniques used and their effectiveness in excluding PDA aneurysms due to MAL syndrome. This single-center retrospective study done at the Dijon-Bourgogne University Hospital included all patients treated by TAE in 2010–2022 for ruptured or unruptured high-flow PDA aneurysms caused by MAL syndrome. We identified 14 patients (7 women and 7 men; mean age, 64 years). Packing and trapping techniques were used alone or together. Occlusion was with microcoils, co-polymer, or cyanoacrylate glue, used separately or combined. Technical success was achieved in 13 (93%) patients. Clinical success was achieved in 12 (86%) patients. One major and two minor complications were recorded within the first 30 days. No complications occurred after 30 days. Follow-up ranged from 1 to 84 months. No cases of aneurysm recanalization have been recorded to date. TAE had high technical and clinical success rates in our patients with unruptured or ruptured PDA aneurysms due to MAL syndrome.

## 1. Introduction

Splanchnic-artery aneurysms are relatively rare, with an incidence of 0.1% to 2% in the general population. Among them, 2% and 1.5% are located on the pancreaticoduodenal arteries (PDA) and gastroduodenal arteries, respectively [1,2,3]. False aneurysms are more common than true aneurysms and may complicate pancreatitis, pancreatic trauma, infection, and surgery [4]. They will not be discussed here, as they differ substantially from true aneurysms [1].

True PDA aneurysms may be associated with celiac-artery occlusion secondary to atherosclerosis, fibromuscular dysplasia, or compression by the median arcuate ligament (MAL) [5,6]. Celiac-trunk stenosis due to extrinsic compression by the MAL, known as MAL syndrome, occurs in about 4% of the general population and is usually an incidental discovery. MAL syndrome seems independent from age, sex, and presence of calcified aortic plaque. Celiac-trunk compression increases the retrograde blood flow from the superior mesenteric artery (SMA) to the celiac artery via the posterior and anterior PDA collaterals, thereby promoting the development of high-flow aneurysms [6,7,8,9].

Several studies suggest that the risk of PDA-aneurysm rupture may be independent from patient age and from aneurysm size, number, and cause. Reported mortality rates after rupture range from 20% to 50% [3,10,11,12,13]. Previous work emphasizes the importance of recognizing and treating PDA aneurysms as quickly as possible [4,14,15].

PDA aneurysms can be treated surgically or by a variety of transarterial embolization (TAE) techniques. A review of the literature until 2002 identified 37 patients with PDA aneurysms, of whom 2 were left untreated due to patient refusal or comorbidities, 23 were treated surgically, and 12 were managed by TAE [16]. No new cases of surgical treatment occurred after 1999. Over the last two decades, TAE has become the reference standard treatment of PDA aneurysms due to its high success rate and lower mortality compared to surgery [17]. However, few data are available on the TAE techniques used and on patient outcomes after TAE for ruptured or unruptured PDA aneurysms.

The objective of this study was to describe the TAE techniques used and their effectiveness in excluding PDA aneurysms due to MAL syndrome. To this end, we evaluated our experience acquired over 12 years at a highly experienced interventional radiology unit in a university hospital.

## 2. Materials and Methods

### 2.1. Population

Consecutive patients admitted to the Dijon-Bourgogne University Hospital (Dijon, France) between January 2010 and May 2022 and treated by TAE for ruptured or unruptured PDA aneurysms due to MAL syndrome were identified retrospectively. Inclusion criteria were celiac-trunk stenosis greater than 50% and one or more PDA aneurysms eligible for TAE. 

Exclusion criteria were false aneurysm, true aneurysm without celiac-trunk stenosis, and celiac-trunk stenosis due to a cause other than compression by the MAL.

### 2.2. The Transcatheter Arterial Embolization Procedure

All TAE procedures were performed by two radiologists with more than 10 years of experience in TAE. Pain control was by local puncture-site anesthesia and mild sedation. The Seldinger technique with insertion of a 6 French (Fr) introducer into the right femoral artery was used in all patients. The first step was celiac-trunk catheterization with a 5-Fr catheter (Terumo, Tokyo, Japan) inserted through the 6-Fr introducer. Competitive flow in the PDA from the SMA due to celiac-trunk stenosis was documented at this step. However, in seven patients, the celiac trunk was completely occluded by the MAL and could not be catheterized. The second step was insertion of a catheter into the SMA and supra-selective catheterization of the afferent aneurysmal artery using a 2.4-Fr Progreat microcatheter (Terumo). 

The operator then chose between, or combined, two techniques, depending on the presentation of the aneurysm and clinical circumstances. In the trapping (or sandwich) technique, controlled-detachable non-fibered microcoils are released to embolize the vessels on either side of the aneurysm [18]. This procedure has the disadvantage of sacrificing the parent artery. The other method, known as the packing technique, consists in super-selective embolization of the aneurysmal sac with microcoils of decreasing size and a liquid embolic agent such as copolymer or biological cyanoacrylate glue, while maintaining permeability of the parent artery [18,19,20]. When the condition was life-threatening, glue was preferred, allowing fast embolization. When a combination of coils and liquid was needed or when a balloon-occlusive microcatheter was needed, the use of a copolymer was preferred, allowing a safer embolization thanks to higher viscosity, whatever the potential duration and radiation dose of the intervention.

A final angiogram was obtained to confirm total exclusion of the aneurysm and to check the patency of the SMA and of the celiac-trunk branches, including the hepatic artery. No anticoagulant or antiplatelet treatment was given following the procedure.

### 2.3. Outcomes and Follow-Up

Technical success was defined as total exclusion of the aneurysm, with no intra-aneurysmal flow after opacification of the afferent artery during the final angiogram. Computed tomography (CT) or magnetic resonance imaging (MRI) with intravenous contrast injection and standard protocols including arterial-phase image acquisition was performed at least one month after TAE; some patients underwent several other imaging studies during their follow-ups. 

Clinical success was defined as the absence of symptoms in previously asymptomatic patients and as the resolution of clinical and laboratory abnormalities in patients with aneurysmal rupture responsible for symptoms (e.g., abdominal pain and/or hemorrhagic shock).

Complications were defined as minor if they had no impact on hospital stay length. Major complications required either hospitalization of previously discharged patients or prolongation of hospitalization in non-discharged patients. Early complications were defined as occurring within the first 30 days after TAE and late complications as occurring after day 30. 

### 2.4. Data Analysis

The study variables were described as number (%) or mean ± SD depending on distribution. 

## 3. Results

### 3.1. Study Population

We included 14 patients (7 women and 7 men) with a mean age of 64.0 ± 13.2 years (range, 35–82 years). Their main characteristics extracted from our hospital database are reported in Table 1.

None of the patients had a known vascular disease before TAE. In one patient, giant-cell arteritis was diagnosed a few weeks after TAE. The main comorbidities were controlled hypertension (n = 5, 36%), current smoking (n = 3, 21%), type 2 diabetes (n = 2, 14%), and dyslipidemia (n = 2, 14%). Mean body mass index was 24.2 ± 4.7 (range, 15.0–33.0). One patient (#8) had cirrhosis of the liver and presented with acute alcoholic pancreatitis. In another (#4), the aneurysm was discovered during evaluation for a liver tumor, which turned out to be a hepatocellular carcinoma. Finally, in another patient (#13), the aneurysm was found by investigations done for a follow-up of colon cancer with liver metastases.

**Figure 1:**
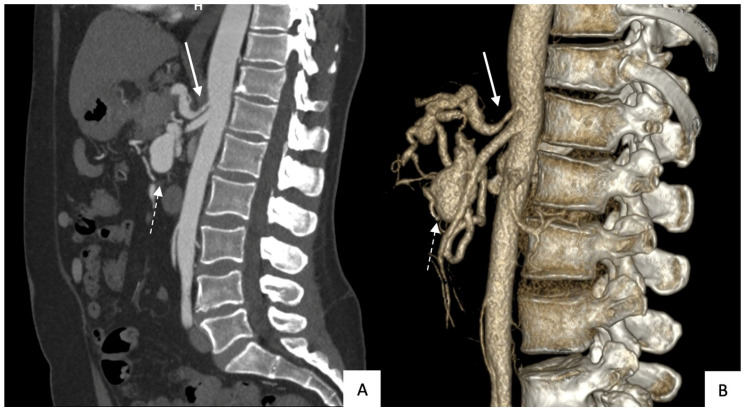
Computed tomography in a sagittal reconstruction (**A**) and with volume rendering (**B**) showing stenosis of the celiac-trunk ostium due to compression by the median arcuate ligament (solid arrow) and an aneurysm of the pancreaticoduodenal artery (dotted arrow).

**Figure 2:**
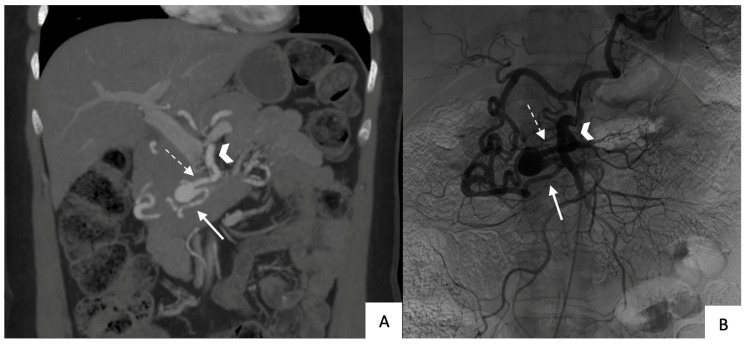
Patient with an unruptured pancreaticoduodenal artery aneurysm: (**A**) Computed tomography in a coronal view with maximum-intensity-projection reformatting showing the aneurysm (arrowhead: superior mesenteric artery; dotted arrow: afferent branch; solid arrow: efferent branch); (**B**) superior mesenteric artery angiogram in the same patient (same annotation).

All patients underwent contrast-enhanced CT with multiplanar-reformation and maximum-intensity-projection reconstructions before TAE to accurately determine the location, size, and shape (saccular or fusiform) of the aneurysm and to determine whether rupture had occurred (Figure 1 and Figure 2). All 14 aneurysms were saccular. Table 1 reports data on size, location, circumstances of the diagnosis, and celiac-trunk stenosis. Thirteen patients each had a single aneurysm. The anterior or posterior branch of the inferior PDA was affected in 79% of cases.

### 3.2. Transcatheter Arterial Embolization

Table 2 provides information on the operating time, fluoroscopy duration, dosimetry, and embolization technique.

Packing was performed alone in three (21%) patients, using only coils (#5, unruptured), only co-polymer (#9, ruptured), or only cyanoacrylate (#10, ruptured), respectively. The coils were controlled-detachable non-fibered microcoils with diameters of 5–20 mm (Concerto, Medtronic, Minneapolis, MN) or 20 mm (Retracta, Cook Medical France, Charenton Le Pont, France), deployed over a 0.035-inch guidewire. The co-polymer was 34% Onyx^®^ (LES, Covidien, Plymouth, MN), an elastic compound composed of ethylene-vinyl-alcohol co-polymer dissolved in dimethyl sulfoxide and mixed with micronized tantalum powder. For this patient (#9, ruptured aneurysm), a balloon catheter was placed into the afferent artery. With the balloon occluded, Onyx^®^ was injected slowly via the catheter until it entirely filled the aneurysmal sac. Finally, for the remaining patient, cyanoacrylate glue (n-butyl cyanoacrylate metacryloxysulfolane, Glubran^®^2; GEM, Viareggio, Italy) mixed with ultrafluid ethiodized alcohol (Lipiodol^®^; Ultra Fluid, Guerbet, Villepinte, France) in a 1:1 ratio was used because of life-threatening bleeding. The aneurysmal sac and parent postero-inferior PDA were occluded with the glue. 

Trapping was used alone in four (29%) patients. In three of these patients (#6 and #7, unruptured; and #12, ruptured), only microcoils were injected. First, controlled-detachable non-fibered microcoils (Concerto, Medtronic; Ruby^®^ Coil, Penumbra, Alameda, CA, USA) were used to ensure good anchorage in the efferent artery, thereby minimizing the risk of downstream migration. Then, non-detachable fibered microcoils (Nester, Cook Medical France) 20 or 40 mm in diameter were injected to complete occlusion of the aneurysmal sac and, if necessary, of the afferent artery. In the remaining patient (#14, ruptured), the catheter could not be passed downstream of the aneurysm and occlusion was therefore achieved using only a liquid co-polymer (SquidPeri18^®^, BALT Group, Montmorency, France).

**Figure 3:**
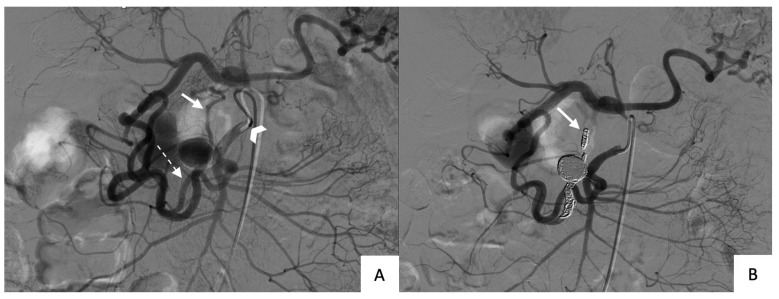
Angiogram of the superior mesenteric artery during transarterial embolization by combined packing and trapping techniques: (**A**) Screening angiogram before treatment (arrowhead: catheter within the superior mesenteric artery ostium; dotted arrow: afferent branch; solid arrow: efferent branch); (**B**) Final angiogram after treatment showing absence of flow into, and downstream of, the efferent branch (solid arrow) compared to the initial angiogram.

Finally, both packing and trapping were used in seven (50%) patients (Figure 3). Microcoils were used alone in three of these patients (#1, #2, and #13; all unruptured), and both microcoils and co-polymer (34% Onyx^®^, LES, Covidien) in four patients (#3, #4, and #8, unruptured; and #11, ruptured).

### 3.3. Outcomes

Results of the TAE procedures are presented in Table 3.

#### 3.3.1. Technical Success

The final angiogram showed complete aneurysm exclusion in 13 (93%) patients. TAE failed in the remaining patient (#12), who was treated for hemorrhagic shock due to aneurysmal rupture. Catheterization of the parent artery was not feasible, and embolization of the gastroduodenal artery failed to exclude the aneurysm. This patient died of hemorrhagic shock after celiac-trunk ostium dissection, on day 15 of the hospital stay. 

In 12 (86%) patients, the parent artery was occluded by the embolization material. One of the patients managed with packing only (#5) had a persistently patent parent artery.

#### 3.3.2. Clinical Success

In addition to patient #12 with failed TAE, one other patient died (#10), on day 1 of the hospital stay, after rupture of an 8-mm aneurysm responsible for severe hemodynamic instability that required emergency packing with cyanoacrylate glue. 

The remaining 12 patients were alive at the last follow-up, with no aneurysm-related symptoms, yielding a clinical success rate of 86% (12/14 patients).

#### 3.3.3. Complications

One major and two minor complications of TAE occurred within the first 30 days. 

About the major complication: in 1 patient (#4), two microcoils initially placed in the afferent aneurysmal artery were carried by the high flow via the PDAs then gastroduodenal arteries into the right branch of the hepatic artery, causing septic necrosis of the right lobe of the liver. This patient recovered with antibiotic therapy. 

About the minor complication: two other patients experienced vascular dissection, of the right iliac artery (#11) and celiac-trunk ostium (#12), respectively. Patient #11 recovered, was discharged on day 7, and had no evidence of further complications on follow-up imaging studies. Patient #12 died on day 15, as described above. 

#### 3.3.4. Other Outcomes

Mean hospital stay length in the nine patients whose aneurysms were asymptomatic was 3.0 ± 4.8 days (range, 0–16 days). In one of these patients (#4), coil migration resulted in prolongation of the hospital stay to 16 days. The range in the other six patients was 0 to 4 days, and three patients were discharged on the day of TAE.

Of the 12 survivors, 1 (#3) was lost to follow-up after the procedure. In two patients, follow-up since TAE was less than one month at the time of this writing. The remaining nine patients underwent CT or MRI at least one month after the procedure. The longest follow-up was 84 months (range, 1–84 months; median, 6 months). None of the follow-up imaging studies showed evidence of aneurysm recanalization or recurrence.

## 4. Discussion

This retrospective review identified 14 patients with PDA aneurysms caused by MAL syndrome and managed by TAE over a 12-year period in a single, highly experienced, interventional-radiology department. Among them, five were treated on an emergency basis after aneurysmal rupture, including two who died of complications from hemorrhagic shock and three who recovered. One patient presented with abdominal pain. Of the remaining eight patients who had no symptoms related to the aneurysm, five had their aneurysm discovered fortuitously and three during evaluation for another condition. TAE had very high technical and clinical success rates, of 93% and 86%, respectively. The two patients who died presented with severe hemorrhagic shock due to aneurysmal rupture. The complications of TAE consisted of celiac-trunk dissection in one of the patients who died and of right-iliac-artery dissection and coil migration, respectively, in two survivors.

True PDA aneurysms account for only about 2% of all splanchnic aneurysms, and those caused by compression of the celiac trunk by the MAL are even less common [1,2,3]. The MAL is a band of fibrous tissue that connects the diaphragmatic crura surrounding the aortic hiatus and is usually situated above the celiac-trunk ostium. However, 10% to 24% of individuals have a low riding MAL that puts pressure on the root of the celiac trunk, which is thus variably narrowed [8,21,22]. This stenosis may increase blood flow in the pancreatic arterial network due to collateral supply from the SMA, potentially causing formation of an aneurysm [2,9,23,24]. Dilation of the peripancreatic arteries has been reported in 80% of patients with celiac-trunk stenosis [25].

Although PDA aneurysms due to MAL syndrome are less common than those related to atherosclerosis, the risk of life-threatening rupture is just as high. Unlike other splanchnic-artery aneurysms, size does not correlate with the risk of rupture. Thus, in one study, the mean size of ruptured PDA aneurysms was only 4.6 mm (range, 2–10 mm) [26]. In the absence of rupture, the diagnosis is often fortuitous, although some patients present with abdominal pain or other symptoms. 

In the past, surgery consisting of ligation, resection, exclusion, or endoaneurysmorrhaphy was the standard reference treatment. However, these procedures carry high morbidity and mortality rates [27]. Advances in imaging and intervention-radiology techniques have allowed the development of TAE as a safer alternative to surgery. Our findings support the use of TAE as the preferred treatment for PDA aneurysms due to MAL syndrome, for several reasons. The technical and clinical success rates in the nine patients without aneurysmal rupture were 100%. These results are consistent with earlier studies reporting no recurrences after TAE [13,23,26,27]. Of these nine patients, four were discharged on the same day and three on the day after TAE, reflecting the limited invasiveness of the procedure, with less anesthetic exposure compared to surgery. It must be noted, however, that three of these nine patients did not have their first follow-up CT or MRI scans to assess for recanalization or recurrence, two because their TAE procedures were too recent at the time of writing, and one who was lost to follow-up. The nine patients with follow-up imaging studies had no evidence of recanalization or recurrence.

The treatment of celiac-trunk stenosis remains controversial, and no clear consensus exists for patients with PDA aneurysm. Given the absence of reported aneurysm recurrences, celiac-trunk revascularization procedures may be best reserved for those patients who have symptoms due to adverse effects on the hepatic blood supply [26,27]. Some groups, however, have advocated celiac-trunk revascularization to prevent recurrence by reestablishing the normal pancreatic arterial circulation [16,28,29]. In that case, surgery should be the preferred approach by section of the median arcuate ligament or bypass [16]. Indeed, stenting of the celiac trunk in such a non-atherosclerotic setting is at high risk of in-stent fracture and/or occlusion, and should be avoided [28,29]. 

Furthermore, the risk of liver or bowel ischemia, or other complications such as pancreatitis, due to embolization is almost nil in such a setting given the important collaterality in this territory and the presence of several pancreaticoduodenal arcades usually, making the sacrifice of one pancreaticoduodenal artery by embolization very safe, even when liquids such as glue are needed. We have never used peripoerative hemodynamic monitoring of the hepatic artery during embolization, as previously described, thinking it is not necessary [20,30,31]. 

Treatment of pancreaticoduodenal artery aneurysms is difficult, especially in patients with multiple aneurysms. Successful treatment of these lesions requires establishing blood flow in the celiac trunk artery region and selecting which aneurysm(s) to treat. In the case of complex anatomy and multiple aneurysms, we always treat first by endovascular approach the ruptured ones in symptomatic patients, and at least the biggest ones in asymptomatic patients, the smallest ones being usually under surveillance [31]. A percutaneous approach can also be proposed in some situations by direct puncture and liquid injection under CT scan. The use of neuro microcatheters, which allows a better endovascular navigability, has made endovascular treatment easier, even in difficult cases. However, a surgical approach may be proposed in selected cases despite a potential higher morbidity rate. Blood flow in the celiac artery region can be established by bypassing the splenic artery and by anastomosing it either directly to the left renal artery or to the abdominal aorta using a graft, as previously described in patients with unruptured pancreaticoduodenal artery aneurysms [32].

The limitations of our study include the retrospective design, which may have resulted in data-retrieval bias. Moreover, the single-center recruitment resulted in a small sample size and may have induced selection bias. We did not compare TAE to surgery. However, TAE is now the reference standard treatment for PDA aneurysms. None of our patients received treatment for the celiac-trunk stenosis, precluding an evaluation of TAE effects on hemodynamics within the pancreaticoduodenal arcades. Finally, follow-up to document the continuing absence of symptoms and the absence of recanalization and recurrence on imaging studies was short. 

## 5. Conclusions

In patients with high-flow PDA aneurysms, TAE may be the treatment of choice both to prevent rupture and to stop bleeding after rupture. When the celiac-trunk stenosis is left untreated, flow remains high within the PDAs. Consequently, longer follow-ups are needed to collect data on the risk of recanalization and the development of new aneurysms. It may be of interest to assess all patients treated for PDA due to MAL syndrome using TAE or surgery, in order to compare these two strategies.

## Figures and Tables

**Table 1:** Characteristics of the 14 patients who underwent transarterial embolization for pancreaticoduodenal artery aneurysm (PDAa).

Patient #	Sex	Age (y)	BMI	Finding	Location of PDAa	Size of PDAa (mm)	Celiak Trunk Stenosis
1	F	73	NA	Fortuitous	Inferior PDA	21	Occluded
2	F	77	21	Fortuitous	Inferior PDA	16	Occluded
3	F	48	28	Fortuitous	Posterior PDA	31	>70%
4	M	68	27	Fortuitous during HCC staging	Antero-inferior PDA	25	>70%
5	M	35	19	Fortuitous	Postero-inferior PDA	20	Occluded
6	F	79	25	Abdominal pain	Inferior PDA	12	Occluded
7	F	50	28	Fortuitous	Inferior PDA	19	Occluded
8	M	66	29	Fortuitous during evaluation of acute pancreatitis	Antero-superior PDA	15	>70%
9	M	56	33	Rupture	Postero-inferior PDA	13	>70%
10	M	73	15	Rupture	Postero-inferior PDA	8	>70%
11	M	70	21	Rupture	Antero-inferior PDA	15	>70%
12	M	82	24	Rupture	Anterior PDA	9	Occluded
13	F	52	20	Fortuitous	Postero-inferior PDA	20	Occluded
14	F	66	24	Rupture	Antero-inferior PDA	8	>50%

PDA: pancreaticoduodenal; PDAa: PDA aneurysm; BMI: body mass index; M: male; F: female; HCC: hepatocellular carcinoma.

**Table 2:** Technical details on the transarterial embolization procedure.

Patient #	Operating Time (min)	Fluoroscopy Duration (min)	Dosimetry (mGy·cm^2^)	Embolization Technique	Embolization Material
1	91	NA	NA	Packing then trapping	DMC
2	60	22	94,749	Packing then trapping	DMC
3	98	40	289,986	Packing then trapping	DMC and Co-polymer
4	129	57	646,030	Packing then trapping	DMC and Co-polymer
5	109	43	227,922	Packing	DMC
6	135	24	312,666	Trapping	DMC
7	150	44	295,857	Trapping	DMC
8	183	71	769,984	Packing then trapping	DMC and Co-polymer
9	285	107	380,047	Packing	Co-polymer
10	95	11	47,624	Packing	Cyanoacrylate
11	165	49	205,835	Packing then trapping	DMC and Co-polymer
12	210	61	596,651	Trapping	DMC
13	115	18	121,123	Packing then trapping	DMC
14	240	41	100,063	Trapping	Co-polymer

NA: not available; DMC: detachable mechanical microcoils.

**Table 3:** Outcomes of transarterial embolization.

Patient #	Final Angiogram	Hospital Stay Length (days)	Time to Last Imaging Study Confirming Exclusion (months)	Early Complications (≤30 days)	Late Complications (>30 days)
1	Exclusion	4	12	None	None
2	Exclusion	4	84	None	None
3	Exclusion	2	Lost to follow-up	None	None
4	Exclusion	16	12	Coil migration	None
5	Exclusion	0	1	None	None
6	Exclusion	0	6	None	None
7	Exclusion	0	2	None	None
8	Exclusion	1	1	None	None
9	Exclusion	1	1	None	None
10	Exclusion	1	Not applicable	Died on day 1	Not applicable
11	Exclusion	7	18	RIA dissection	None
12	Failure	15	Not applicable	Celiac-trunk ostium dissection Death on day 15	Not applicable
13	Exclusion	0	Insufficient follow-up ^a^	None	None
14	Exclusion	10	Insufficient follow-up ^a^	None	None

RIA: right iliac artery; ^a^ these two patients were treated less than one month before the writing of the study report.

## Data Availability

The data presented in this study are available on request from the corresponding authors. The data are not publicly available due to identity reasons.
